# Concentration Kinetics of Serum MMP-9 and TIMP-1 after Blunt Multiple Injuries in the Early Posttraumatic Period

**DOI:** 10.1155/2012/435463

**Published:** 2012-03-27

**Authors:** M. Brumann, T. Kusmenkov, L. Ney, K.-G. Kanz, B. A. Leidel, P. Biberthaler, W. Mutschler, V. Bogner

**Affiliations:** ^1^Chirurgische Klinik und Poliklinik-Innenstadt, Ludwig-Maximilians University, 80336 Munich, Germany; ^2^Klinik für Anästhesiologie, Ludwig-Maximilians University, 80336 Munich, Germany; ^3^Department of Emergency Medicine, Campus Benjamin Franklin, Charité-University Medical Centre, 12203 Berlin, Germany; ^4^Klinik und Poliklinik für Unfallchirurgie, Klinikum Rechts der Isar, Technische Universität München, 81675 Munich, Germany

## Abstract

Metalloproteinases are secreted in response to a variety of inflammatory mediators and inhibited by tissue inhibitors of matrixmetalloproteinases (TIMPs). Two members of these families, MMP-9 and TIMP-1, were differentially expressed depending on clinical parameters in a previous genomewide mRNA analysis. The aim of this paper was now to evaluate the posttraumatic serum levels and the time course of both proteins depending on distinct clinical parameters. 60 multiple traumatized patients (ISS > 16) were included. Blood samples were drawn on admission and 6 h, 12 h, 24 h, 48 h, and 72 h after trauma. Serum levels were quantified by ELISA. MMP-9 levels significantly decreased in the early posttraumatic period (*P* < 0.05) whereas TIMP-1 levels significantly increased in all patients (*P* < 0.05). MMP-9 and TIMP-1 serum concentration kinetics became manifest in an inversely proportional balance. Furthermore, MMP-9 presented a stronger decrease in patients with severe trauma and non-survivors in contrast to minor traumatized patients (ISS ≤ 33) and survivors, initially after trauma.

## 1. Introduction

Posttraumatic immune activation and dysfunction in form of systemic inflammation response syndrome (SIRS), subsequent multiple organ dysfunction syndrome (MODS), and multiple-organ failure (MOF) still remains a leading cause of late posttraumatic morbidity and mortality [1]. MOF exerts a profound influence on patient outcome, as it occurs in one-fourth of all patients suffering from blunt multiple injuries and accounts for 27.5% of death among trauma patients [2].

Previous genomewide studies have linked specific mRNA expression patterns in monocytes with adverse outcome. Among these differentially expressed genes that were significantly connected to distinct clinical parameters like injury severity or outcome, matrix metalloproteinase-9 (MMP-9) and its specific tissue inhibitor-1 (TIMP-1) could be identified to play an important role in trauma patients in the early posttraumatic period [3].

MMP-9 is part of the matrix metalloproteinase (MMPs) family presenting a group of genetically distinct, but structurally related zinc-containing proteolytic enzymes. Collectively, MMPs play a central role in tissue remodeling of extracellular matrix (ECM) being capable of degrading all kinds of matrix components. Participating in ECM degradation they are involved in many biological processes such as embryogenesis, angiogenesis, and wound healing [4]. They are secreted as nonactive proenzymes in response to a variety of inflammatory mediators and they are inhibited in vivo by TIMPs [5]. Under physiological conditions MMP activities are precisely regulated at several levels like transcription or precursor zymogen activation and inhibition by their specific endogenous inhibitors.

MMP-9 (92 kDa gelatinase) is a type IV collagenase implicated in various aspects of inflammation including accumulation of inflammatory cells, healing of tissue injury, and remodeling processes. Specifically, it has been shown to mediate vascular leakage and to initiate the migration of inflammatory cells inducing wound repair.

Furthermore, it is stored in the tertiary granules of polymorphonuclear leucocytes, which are key effectors in acute inflammatory diseases. Various cell lines such as keratinocytes, eosinophils, neutrophils, and macrophages can also express MMP-9 [6].

TIMP-1 works as a natural inhibitor of MMP-9 and is found in most tissues and body fluids. By inhibiting MMPs activities, TIMPs are involved in tissue remodeling and regulation of ECM metabolism. The TIMP family consists of four members sharing important structural features as well as the ability of MMP inhibition. Under normal physiological conditions, TIMPs bind MMPs in a 1 : 1 stoichiometry [4].

Consequently, a loss of activity control may result in a variety of diseases such as arthritis, cancer, arteriosclerosis, and fibrosis. Thus, the balance of MMP and TIMP activities plays the pivotal role in both physiological and pathological events [4].

Neither MMPs nor TIMPs have been described in patients suffering from multiple major trauma and subsequent posttraumatic immune system alterations.

In dependence on the persuasive results of a serial screening analysis of monocyte mRNA expression patterns after blunt multiple injuries, main intention of this investigation was the try to reproduce the MMP-9 and TIMP-1 transcriptional profiles of an immune cell fraction (monocytes) in serum samples of major trauma patients and their potential relationship to distinct clinical parameters.

## 2. Patients and Methods

In this study, performed at a Level I trauma center according to Good Clinical Practice (GCP), 60 adult patients (age > 18 years) presenting with multiple injuries and Injury Severity Score (ISS) of greater than 16 points were included. Patients were enrolled if the emergency department was reached within 90 minutes after the traumatic event.

Signed informed consent was obtained from each patient or their legal representatives over the course of time. Ethical Committee Permission was obtained from the Ludwig-Maximilians University, Munich, Germany (reference number: 012/00). Patients who did not survive the first 24 hours after trauma were excluded.

After initial resuscitation and/or primary surgical intervention according to standard of care, patients were admitted to intensive care unit.

Retrospectively, patients were distributed to different groups in regard to the two following clinical parameters: injury severity assessed by ISS according to AIS98 and outcome expressed by the survival of 90 days after trauma.

Serum samples were collected on admission (0 h), 6 h, 12 h, 24 h, 48 h, and 72 h after trauma. Afterwards they were stored at −80°C.

Protein concentrations were quantified by enzyme linked immunosorbent assay (ELISA; Human-MMP-9 and Human-TIMP-1 ELISA, Bender MedSystems GmbH).

For the analysis of MMP-9 and TIMP-1 protein levels in serum, sample dilutions of approximately 1 : 100 to 1 : 500 for MMP-9 and 1 : 1000 to 1 : 5000 for TIMP-1 were required to minimize the numbers of non evaluable results.

Resulting data was statistically analyzed using Mann-Whitney Rank Sum Test (SigmaStat/SigmaPlot). A *P* value of *P* < 0.05 was considered significant.

## 3. Results

### 3.1. Patient Collective and Clinical Data

Altogether 60 patients fulfilled the entry criteria and were included into the study. Patients who did not reach the emergency department within 90 minutes after trauma, patients who were younger than 18 years, and patients who were pregnant or under guardianship were excluded.

There were 42 male and 18 female major trauma patients (mean age: 45 years; range: 18 to 93 years).

Patients were separated into different groups according to injury severity and outcome. Injury severity was coded according to ISS. We separated the patient collective into two groups regarding the severity of trauma; that is, we used a cut-off value of 33 points (group 1a: ISS ≤ 33, *n* = 33; group 1b: ISS > 33, *n* = 27). Median ISS in all patients was 33 (SD 11; range: 17 to 66). Median ISS of group 1a was 27 points (SD 5); median ISS of group 1b was 50 points (SD 22). Outcome was defined as a survival of 90 days after trauma. Six patients deceased during this time frame (group 2a: survivors, *n* = 54; group 2b: non-survivors, *n* = 6).

Six patients did not survive the traumatic event (mean age of non-survivors: 54 years; range: 37–93 years).

### 3.2. Serum Protein Concentrations

#### 3.2.1. Time Course

**Figure 1:**
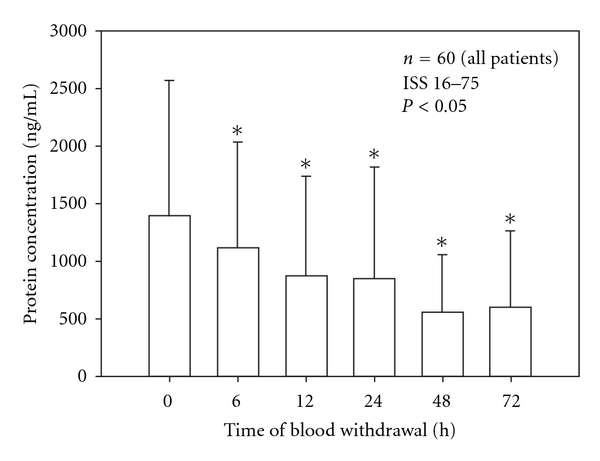
Significant decrease of mean MMP-9 serum levels in the early posttraumatic period in comparison to initial mean MMP-9 serum concentrations at 0 h after the traumatic event. Data were analyzed by Mann-Whitney Rank Sum test and presented by mean ± standard error of the mean (*P < 0.05).

**Figure 2:**
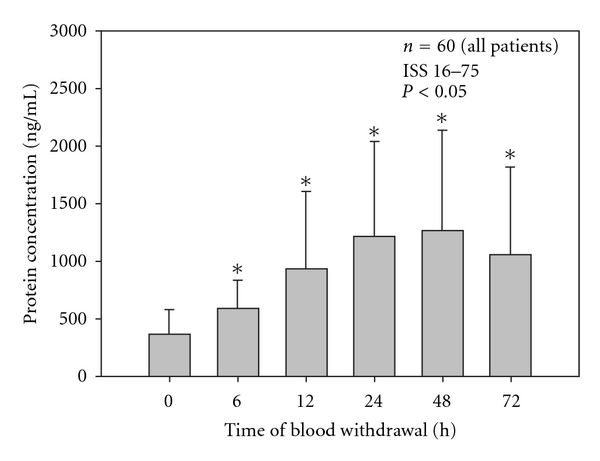
Significant increase of mean TIMP-1 serum concentration levels 6 h–72 h after the traumatic event compared to initial mean TIMP-1 serum level at 0 h after trauma. Data were analyzed by Mann-Whitney Rank Sum test and presented by mean ± standard error of the mean (*P < 0.05).

Mean serum levels of MMP-9 and TIMP-1 were detected by immunosorbent assay in a multiple-trauma patient collective in the early posttraumatic period.  Figure 1 shows MMP-9 concentration in ng/mL during the time after trauma; Figure 2 demonstrates TIMP-1 concentration in ng/mL over the time. 

MMP-9 reached a high concentration level in all investigated patients initially after trauma at the time point of 0 h. Afterwards, mean serum levels of MMP-9 decreased significantly over the posttraumatic time period (6 h–72 h) compared to serum concentration detected directly after trauma (0 h).

In contrast to that, TIMP-1 started with low serum concentration, which was continuously increasing during this observation period (Figure 2). Mean serum levels of TIMP-1 significantly increased during the observed time period compared to the initial serum level (0 h).

**Figure 3:**
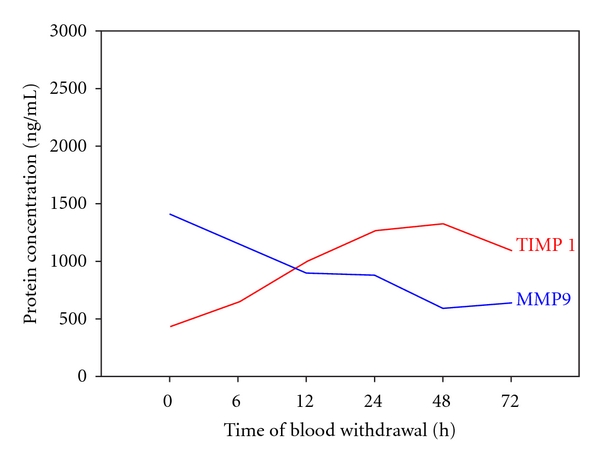
Significant inversely proportional concentration kinetics of MMP-9 and its specific inhibitor TIMP-1 during the observed posttraumatic time period of 72 h. The blue line indicates the connection of all measured time points concerning the significant decrease of MMP-9 serum concentration whereas the red one indicates the connection of all measured time points regarding the significant increase of TIMP-1 serum concentration.

Regarding the characteristics of MMP-9 and TIMP-1 serum levels, Figure 3 combined time course of MMP-9 and its specific inhibitor TIMP-1 during the observed time period of 72 h after trauma. Both proteins demonstrated a significant universal concentration pattern in all investigated patients after severe injury, as MMP-9 showed a significant downregulation whereas its physiological inhibitor TIMP-1 presented a significant upregulation in the posttraumatic period. This study's results show a specific posttraumatic serum concentration kinetics expressed by an inversely proportional balance of MMP-9 and TIMP-1 in the early time frame after multiple injuries.

#### 3.2.2. ISS

**Figure 4:**
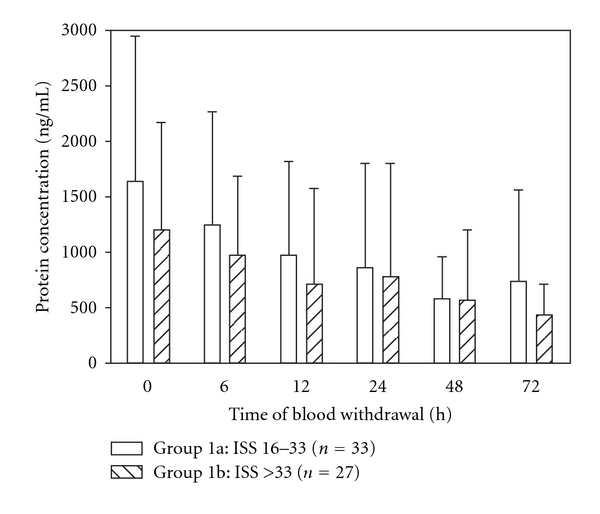
There is a lower serum MMP-9 activity in patients presenting with an ISS > 33 points in contrast to those with an ISS ≤ 33 regarding the first 12 h after trauma. Concerning the other points of time, there is no further remarkable significant difference between group 1a and 1b. Data were analyzed by Mann-Whitney Rank Sum test and presented by mean ± standard error of the mean.

Patients of group 1b presenting with an ISS greater than 33 points showed a remarkable lower mean MMP-9 serum concentration level initially (0 h) and in the following 12 h after major injury than patients of group 1a representing a collective of patients suffering from moderate injury with an ISS between 16 and 33 points (Figure 4). Concerning the following time points, mean MMP-9 serum concentrations of both groups decreased continuously without any remarkable significant difference.

**Figure 5:**
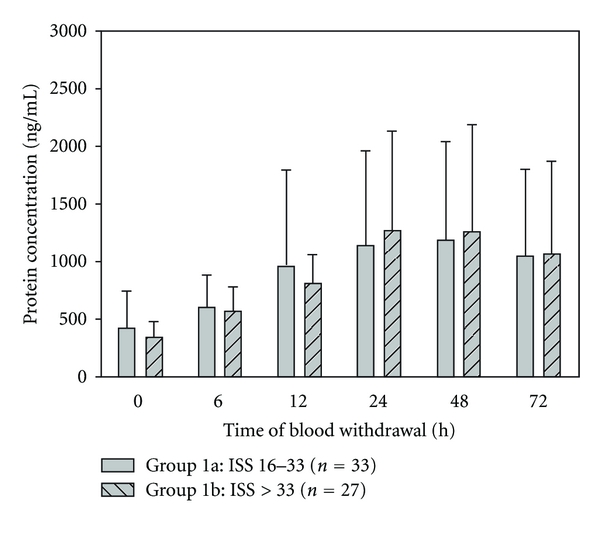
Patients after major trauma (ISS > 33) and patients after moderate injury (ISS ≤ 33) showed nearly equal mean serum levels of TIMP-1 in the early posttraumatic period over 72 hours. There were no significant differences found between these two groups. Data were analyzed by Mann-Whitney Rank Sum test and presented by mean ± standard error of the mean.

Mean TIMP-1 serum concentration levels of both groups did not distinguish significantly (Figure 5). TIMP-1 concentration levels increased in the observed time period of 72 h after trauma in group 1a and 1b without notable difference.

#### 3.2.3. Outcome

**Figure 6:**
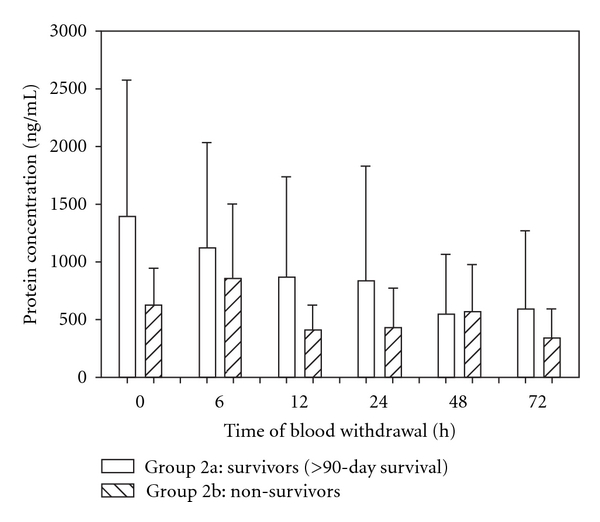
Mean MMP-9 serum levels were remarkably lower in non-survivors (group 2b) than those in survivors, especially within the 24 h after the traumatic event. Data were analyzed by Mann-Whitney Rank Sum test and presented by mean ± standard error of the mean.

Mean MMP-9 serum levels of patients who did not survive the traumatic event (group 2b) during an observation period of 90 days were compared to those who survived (group 2a) in Figure 6. Remarkably, MMP-9 revealed considerable lower mean serum levels in non-survivors (group 2b) than in survivors (group 2a) immediately after multiple major injuries within the first 24 h after the traumatic event (Figure 6). In the subsequent time interval, there were no significant differences between the two groups. All in all, mean MMP-9 serum levels of patients who did not survive the posttraumatic 90-day time frame revealed lower serum protein concentrations than those who survived the traumatic event. 

**Figure 7:**
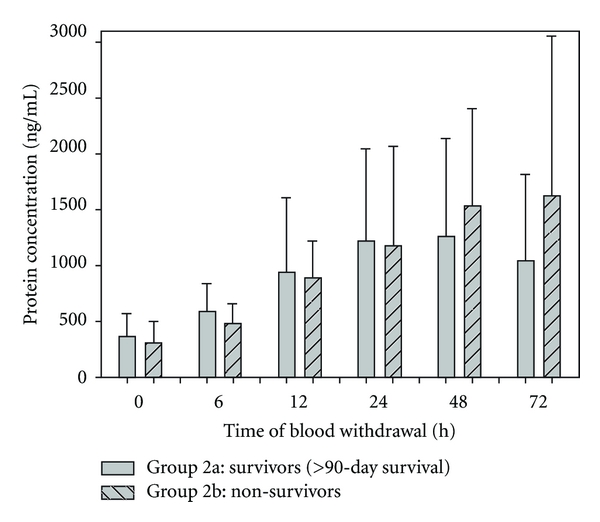
Mean TIMP-1 serum levels were lower in non-survivors (group 2b) than those in survivors (group 2a) within the first 24 h after multiple major injuries. 48 h and 72 h after trauma, mean TIMP-1 serum levels of non-survivors were higher than mean protein levels of patients who survived 90-days after the traumatic event. Data were analyzed by Mann-Whitney Rank Sum test and presented by mean ± standard error of the mean.

In Figure 7, mean TIMP-1 serum levels of survivors (group 2a) and non-survivors (group 2b) are illustrated. Observing the first 24 hours (0 h–24 h), mean TIMP-1 serum levels of non-survivors are lowered in comparison to mean serum TIMP-1 levels of survivors. Regarding TIMP-1 serum levels at the moment of 48 h and 72 h, patients who did not survive after trauma expressed definite higher mean TIMP-1 serum levels.

## 4. Discussion

A precedent serial, sequential oligonucleotide microarray analysis of initial posttraumatic monocyte messenger RNA could evaluate differential expression profiles in patients suffering from multiple injuries. Thereby, specific gene expression profiles have been identified to be significantly associated with important clinical parameters like injury severity and outcome [3].

Two of those identified eligible genes that were differentially expressed according to clinical parameters code for the two proteins were investigated in this study: MMP-9 and its specific endogenous inhibitor TIMP-1.

Furthermore, matrix metalloproteinases are regarded to be key role molecules in inflammation [7], as they are involved in pathophysiological remodeling of the vascular wall [8]. The aforementioned results of a previous genomewide mRNA analysis on the one hand and the gained prominence of MMPs as key role effectors in tissue turnover, inflammation, and several diseases on the other hand generated the intention to examine serum MMP-9 and TIMP-1 kinetics, to evaluate their balance and to identify their outcome clarifying potential in patients after multiple major injuries in the early posttraumatic period.

Consequently, the primary aim of this study was to evaluate the balance of these proteins and to reproduce the outcome clarifying monocyte mRNA signature in serum samples of multiple injured patients.

In this context, serum level alterations of MMP-9 and TIMP-1 depending on clinical outcome parameters could have been the answer to early identify patients who are at risk to develop severe posttraumatic complications in response to their posttraumatic immune dysfunction leading to an unfavorable outcome.

Especially, since a genomewide mRNA analysis is particularly expensive, time-consuming, and restricted in terms of a widely spread availability, altered outcome predicting serum levels in multiple trauma patients could offer an instant and easily performable chance to receive an individual view on the immune status of each single patient and thereby evaluate the individual risk of posttraumatic morbidity and mortality.

### 4.1. Protein Serum Levels

#### 4.1.1. Time Course and Balance

The main and most important result of this study is the identification of the convincing universal concentration pattern of MMP-9 and TIMP-1 in all investigated patients in the early posttraumatic period. For the first time, this study's results can state a significant decrease of mean MMP-9 serum levels in the first 72 h after trauma and simultaneously demonstrate a significant increase of its specific inhibitor TIMP-1 in serum of multiple traumatized patients immediately after the traumatic event. Both proteins state an inversely proportional balance after major injury, as it has not been evaluated in any other study so far.

Neither MMP-9 nor TIMP-1 levels have yet been examined in patients suffering from blunt multiple injuries. However, both proteins have built the basic of a multiplicity of studies but have never been described in context of multiple traumas. MMP-9 serum concentration levels have been described in the context of several diseases such as multiple sclerosis [9], coronary artery disease [10], multiple myeloma [11, 12], and chronic lymphocytic leukemia [13, 14]. TIMP-1 serum concentration levels have been examined in patients suffering from several types of cancer such as colorectal cancer [15] and lung cancer [16], and also in terms of acute disseminated encephalitis [17] or liver fibrosis [18].

Regarding the results of this current study, MMP-9 and TIMP-1 present a characteristic concentration pattern and contemporaneously a functional imbalance in patients after multiple injuries, since the concentration kinetics became manifest in an inversely proportional posttraumatic time course. In regard to ISS and MOF, our results demonstrate strong changes of MMP-9 concentration levels, especially in severely injured patients (ISS > 33) and non-survivors initially after trauma within the first 24 h after trauma. This initial downregulation of MMP-9 in these two groups may be understood as an answer to the severity of trauma, due to the fact that it implicates nearly a loss of MMP-9 serum concentration and in this context a loss of remodeling potential.

There might be different conceivable explanations for these phenomena. In healthy subjects, MMP- 9 is cosecreted with TIMP-1 in 1 : 1 stoichiometry. TIMPs are specific inhibitors of MMPs whereupon TIMPs 1–4 can inhibit all MMPs. TIMPs inhibit all MMPs tested so far, except that TIMP-1 fails to inhibit MT1-MMP [19].

Under pathological conditions, which are associated with an imbalance of MMP activities, changes of TIMP levels seem to play an important role as they directly influence the level of MMP activity [4].

Thus, in patients with multiple major injuries, there is a significant overproduction of TIMP-1 over MMP-9 as investigated in this study. The functional consequence of the TIMP-1/MMP-9 imbalance in multiple traumatized patients is impressive and may in this context be understood as a generalized downregulation of MMP-9 activity and consequently its remodeling potential induced by the overexpression of TIMP-1 in the very early posttraumatic period.

Remodeling is defined as to “model again or reconstruct”. As already mentioned, the main role of MMPs remains ECM and basement membrane breakdown in the context of tissue remodeling and angiogenesis, whereas the key role of TIMPs is the maintenance of the essential balance between deposition and degradation of ECM by their function as endogenous inhibitors of MMP activities. The major intention of wound healing is the restoration of a functional connective tissue. Additionally, this regenerative process depends upon the accumulation and deposition of ECM molecules and besides the remodeling of ECM by MMPs [19].

In accordance with these physiological mechanisms, this study's result may be construed in the following way. The significant decrease of MMP-9 in the early posttraumatic period induced by a coincidental, significant overproduction of its physiological inhibitor in all patients after severe injury might reflect the organism's reaction on the distinct tissue injury and immune reaction caused by the traumatic event. MMP-9 activity—if correlated with the “remodeling potential”—has to be reduced significantly to cope with the posttraumatic damage. In consequence, the severely injured organism restricts the regenerative process depending upon MMP activity. This can be understood as a “rescue” response to the impact of trauma and the entailed overwhelming posttraumatic immune alteration.

Nevertheless, these protein serum results depending on injury severity and outcome could only demonstrate tendencies and could not reproduce the highly significant changes in monocyte mRNA expression patterns initially after trauma in a genomewide analysis so far.

There are several suggestions in the literature about monocytes playing a pivotal role in posttraumatic immune alterations, as they are an important part of innate immune system [20]. Hypothetically, the very early monocyte mRNA expression changes cannot be found in serum, as MMP-9 concentration changes once initiated in the intracellular space have not been secreted into serum so far.

On the other hand, monocytes only represent a small cell fraction of the cellular blood ingredients (2–8% of all leucocytes), in comparison to the multiple ways of secretion, activation, and inhibition seen in patients' serum. This may explain why the distinct significance in the monocyte signature cannot be clearly reproduced in serum.

#### 4.1.2. Blood Sampling

The determination of matrix metalloproteinases in peripheral blood as a noninvasive and easily performable possibility of diagnosis and monitoring of several diseases has already been recommended, since MMP activities on cellular level are reliably reflected in body fluids such as serum and plasma [21]. Peripheral blood contains several forms of MMPs. It can be found in soluble constitutive form in plasma and in form of intracellular zymogenes in platelets and leukocytes, which is followed by a release of proteinase into the circulation particularly in terms of a disease [22]. Thus, changes of MMP activity in serum measurements have been detected in several diseases and can therefore act as an indicator for pertinent intracellular protease activity [23].

However, discrepancies in MMP concentration levels depending on blood sampling procedure have been described in several studies [24]. Possible preanalytical errors of TIMP and MMP measurement in peripheral blood have been a matter of debate. In this context, elevated serum MMP levels have been described as serum levels of matrix metalloproteinases are likely influenced by MMP release following degranulation of leukocytes and platelets during the ex vivo blood clotting [25].

Nevertheless, most studies work with serum as medium to evaluate MMP concentration levels, because serum represents a likely affordable and reliable blood sampling procedure to easily investigate MMP and TIMP concentration levels in patients suffering from several diseases. Serum concentrations have been detected in terms of neurodegenerative diseases, cardiovascular diseases, or malignant proliferation, for example [23].

#### 4.1.3. Patient Collective and Clinical Data

In reference to mean age (45 years) and distribution between the sexes of nearly 2 : 1 (men : women), this study's patient collective is well comparable to patient collectives described in assimilable European and American studies of multiple injured patients [26].

In accordance with the aforementioned entry criteria, only patients who reached the emergency department within 90 minutes after trauma were included in this study. In regard to other research groups' investigations on circulating neutrophils, the chosen blood-sampling time points in this study were set in the following way: on admission, which means within 90 minutes after trauma, and again 6 h, 12 h, 24, 48 h, and 72 h after the traumatic event [27].

It is well known that particularly the very early posttraumatic period sets the course for the appearance of a later multiple-organ failure [28]. Furthermore, trauma-induced alterations in the innate immune response lead to a subsequent immune cell-regulated defense reaction ending in hyperinflammation, immunoparalysis, or a combination of both, particularly in the early posttraumatic period [29]; therefore, a serial analysis of inflammation parameters including this vulnerable posttraumatic time frame after multiple major injuries is indispensable.

The definition of injury severity and the development of multiple-organ failure as well as setting the respective cut-off points in order to build dichotomous clinical groups requires the use of scoring systems as it was done in our study using ISS.

For this reason, serum samples were only collected if patients suffered from blunt multiple injuries with an injury severity greater than 16 points, as assessed by the ISS-Scoring System, the standard method of rating severity of injury [30]. In regard to injury severity, a cut-off level had to be defined to differentiate between minor and major traumatized patients. That is why we depended on building two opposite groups for our analysis, as the number of our investigated patient collective was too few to calculate a reliable multiple regressive analyses respecting the particular ISS count in every single patient. For these biostatistical reasons, we switched to the analysis of two dichotomous groups. Concerning injury severity, a cut-off of 33 points was appointed in this study to distinguish between major and minor traumatized patients, for an ISS of 33 points was the median ISS in our patient collective. This might be a limiting factor of this study. However, there is no definitive ISS cut-off level differentiating between minor and major traumatized patients published in literature so far.

In contrast to ISS mentioned previously, outcome is a simple and straightforward clinical criterion as it is just defined as a survival of more than 90 days after trauma. The evident advantage of this clinical criterion is the clear definition whereas its main limitation might be the inhomogeneity exhibited in both groups (survivors, non-survivors) as neither the cause of death nor the variety of posttraumatic individual complications after surviving the initial trauma is attended.

## 5. Conclusions

Regarding the time course of significantly decreasing MMP-9 and significantly increasing TIMP-1 serum levels, we can state a universal concentration pattern after blunt multiple injuries in all investigated patients. Both proteins show a typical inversely proportional serum concentration kinetics in the first 72 h after trauma.

Furthermore, the very early posttraumatic period is characterized by a remarkable decrease of MMP-9 serum concentration initially after trauma in patients with a major injury (ISS > 33) and those who did not survive the traumatic event in contrast to patients with moderate injury and those who survived. This might be due to the fact that the remodeling potential of MMP-9 is strongly downregulated in patients suffering from severe traumatic injury as well as in non-survivors.

The inversely proportional balance between MMP-9 and its endogenous inhibitor TIMP-1 and the decrease of MMP activity in severely injured (ISS > 33) and non-survivors suggest a potentially new mechanism of posttraumatic immune system dysbalance and SIRS/MOF precondition following multiple injuries.
